# Fully automated open‐source lesion mapping of T2‐FLAIR images with FSL correlates with clinical disability in MS

**DOI:** 10.1002/brb3.440

**Published:** 2016-01-28

**Authors:** Nathan C. Wetter, Elizabeth A. Hubbard, Robert W. Motl, Bradley P. Sutton

**Affiliations:** ^1^ Department of BioengineeringUniversity of Illinois at Urbana‐ChampaignUrbanaIllinois; ^2^ Beckman Institute for Advanced Science and TechnologyUniversity of Illinois at Urbana‐ChampaignUrbanaIllinois; ^3^ Department of Kinesiology and Community HealthUniversity of Illinois at Urbana‐ChampaignUrbanaIllinois

**Keywords:** Atrophy, magnetic resonance imaging, multiple sclerosis, outcome measurement, T2 lesions

## Abstract

**Background:**

T2 Lesion Volume (T2LV) has been an important biomarker for multiple sclerosis (MS). Current methods available to quantify lesions from MR images generally require manual adjustments or multiple images with different contrasts. Further, implementations are often not easily or openly accessible.

**Objective:**

We created a fully unsupervised, single T2 FLAIR image T2LV quantification package based on the popular open‐source imaging toolkit FSL.

**Methods:**

By scripting various processing tools in FSL, we developed an image processing pipeline that distinguishes normal brain tissue from CSF and lesions. We validated our method by hierarchical multiple regression (HMR) with a preliminary study to see if our T2LVs correlate with clinical disability measures in MS when controlled for other variables.

**Results:**

Pearson correlations between T2LV and Expanded Disability Status Scale (EDSS:* r* = 0.344, *P* = 0.013), Six‐Minute Walk (6MW:* r* = −0.513, *P* = 0.000), Timed 25‐Foot Walk (T25FW:* r* = −0.438, *P* = .000), and Symbol Digit Modalities Test (SDMT:* r* = −0.499, *P* = 0.000) were all significant. Partial correlations controlling for age were significant between T2LV and 6MW (*r* = −0.433, *P* = 0.002), T25FW (*r* = −0.392, *P* = 0.004), and SDMT (*r* = −0.450, *P* = 0.001). In HMR, T2LV explained significant additional variance in 6MW (*R* ^2^ change = 0.082, *P* = 0.020), after controlling for confounding variables such as age, white matter volume (WMV), and gray matter volume (GMV).

**Conclusion:**

Our T2LV quantification software produces T2LVs from a single FLAIR image that correlate with physical disability in MS and is freely available as open‐source software.

## Introduction

Lesions, or white matter hyperintensities, have long been a defining feature of the clinical diagnosis and tracking of multiple sclerosis (MS), becoming incorporated into the McDonald criteria in the 2010 revisions (Montalban et al. 2010; Polman et al. 2011). Lesions further have been a primary end point of trials examining the efficacy of disease‐modifying therapies in MS (Sormani et al. 2010; Sormani and Bruzzi 2013). Such lesions presumably reflect periods of immune‐mediated disease activity within the CNS (Bjartmar and Trapp 2001; Hemmer et al. 2006; Trapp and Nave 2008).

The identification and measurement of lesions on T2 MRI images has been an important aspect of research in MS, enabling direct viewing of impact on brain tissue through T2 lesion volumes (T2LV) (Filippi et al. 2011). Imaging contrast and lesion delineation have improved with recent advances in MRI acquisition sequences (Miller et al. 2014) to include high resolution, 3D acquisitions of T2‐weighted flow attenuated inversion recovery (FLAIR) imaging, which result in a T2‐weighted image with suppression of CSF according to its T1 constant (Paniagua Bravo et al. 2014). However, reliable and automated methods for quantification of lesions, along with open‐source methods for reproducible research, are severely lacking in this area. This inhibits more direct comparisons of measures across imaging sites or studies.

Several computational methods have been proposed to quantify T2LV, yet they often are proprietary, require human intervention, or require multispectral (or multicontrast) imaging data sets (Mortazavi et al. 2011; Lladó et al. 2012). Our goal was to create robust, fully automated T2LV quantification using an approach that is openly available and easy to use. We have built upon the open‐source image processing toolkit FSL (fMRIB Software Library, fMRIB, Oxford, http://fsl.fmrib.ox.ac.uk/fsl, Smith et al. 2004) to create lesion‐measuring software that requires only the T2 image. We demonstrate the utility of this approach by estimating associations between T2LV and metrics of clinical disability in a group of MS subjects. This tool will benefit research in MS by providing a common approach to quantify lesions and help to make methods and results of studies more comparable with one another.

## Methods

### Subjects

Of 64 total subjects recruited for this study, 52 (11 male, 41 female, see Table 1) completed the imaging study and all clinical disability measures, and remained after exclusion of outliers. The subjects included 40 with relapsing–remitting MS (RRMS), seven with secondary progressive MS (SPMS), and three with primary progressive MS (PPMS). The average age was 51 years and average disease duration was 12 years. MS subtype and disease duration were unavailable for two subjects. Potential subjects were recruited through a database of participants from our previous studies and flyers distributed among patients in the North American Research Committee on Multiple Sclerosis (NARCOMS) registry. The following inclusion criteria were utilized: (1) MS diagnosis, verified by physician; (2) relapse‐free for at least 30 days; (3) age 18–64 years; (4) ambulation with or without assistive device; and (5) physician's approval for participation. All participants provided informed consent in accordance with the Institutional Review Board.

**Table 1:** Descriptive statistics

	N	Min	Max	Mean	SD
T2LV (% Brain Volume)	52	0.01	4.01	0.86	1.01
Age (Years)		25	64	51.0	8.4
WMV (Normalized mm^3^)		566,690	849,671	721,215	56,756
GMV (Normalized mm^3^)		525,955	783,608	662,147	52,034
EDSS (Score)		0.0	7.5	5.5^a^	2.84^a^
6MW (Feet)		47	2479	1131	620
T25FW (Feet/second)		0.37	7.62	3.87	1.92
SDMT		20	77	46.06	12.16
Disease duration (Years)	50	1	29	12.24	8.44
Disease subtype					
RRMS	40				
SPMS	7				
PPMS	3				

T2LV, T2 Lesion Volume; WMV, White Matter Volume; GMV, Gray Matter Volume; EDSS, Expanded Disability Status Scale; 6MW, 6 Minute Walk; T25FW, Timed 25 Foot Walk; SDMT, Symbol Digit Modalities Test; RRMS, Relapsing–Remitting MS; SPMS, Secondary Progressive MS; PPMS, Primary Progressive MS; SD, Standard Deviation.

a For EDSS, median and interquartile range are reported instead of mean and SD.

### MRI acquisition

Volunteer subjects were scanned on a Siemens (Erlangen, Germany) Trio 3 T MRI scanner with a 12‐channel head coil. Two structural acquisitions were used to assess the performance of the T2LV quantification method: a fluid attenuated inversion recovery (FLAIR) sequence to obtain T2LVs, and a magnetization prepared rapid acquisition of gradient echo (MPRAGE) T1‐weighted scan to obtain atrophy measures for comparison. These were standard sequences provided by Siemens. The FLAIR scan was a 3D turbo spin echo (TSE) using a variable flip angle. The scan was used to acquire 1 mm isotropic resolution with coverage of the whole brain in 7 min with a sagittal prescription. Relevant parameters for the FLAIR sequence were 2.2 sec inversion time, TE/TR of 388 msec/6 sec, and parallel imaging with a generalized autocalibrating partial parallel acquisition (GRAPPA) factor of 2 (Griswold et al. 2002). The T1 MPRAGE scan was a 3D gradient echo, using an inversion time of 900 msec. The scan was used to acquire 0.9 mm isotropic resolution with coverage of the whole brain in 4.5 min with a sagittal prescription. Relevant parameters of the T1 MPRAGE sequence are TE/TR of 2.32 msec/1.9 sec, and a parallel imaging acceleration factor of 2.

### Image analysis

#### Quantification of lesion volumes

**Figure 1:**
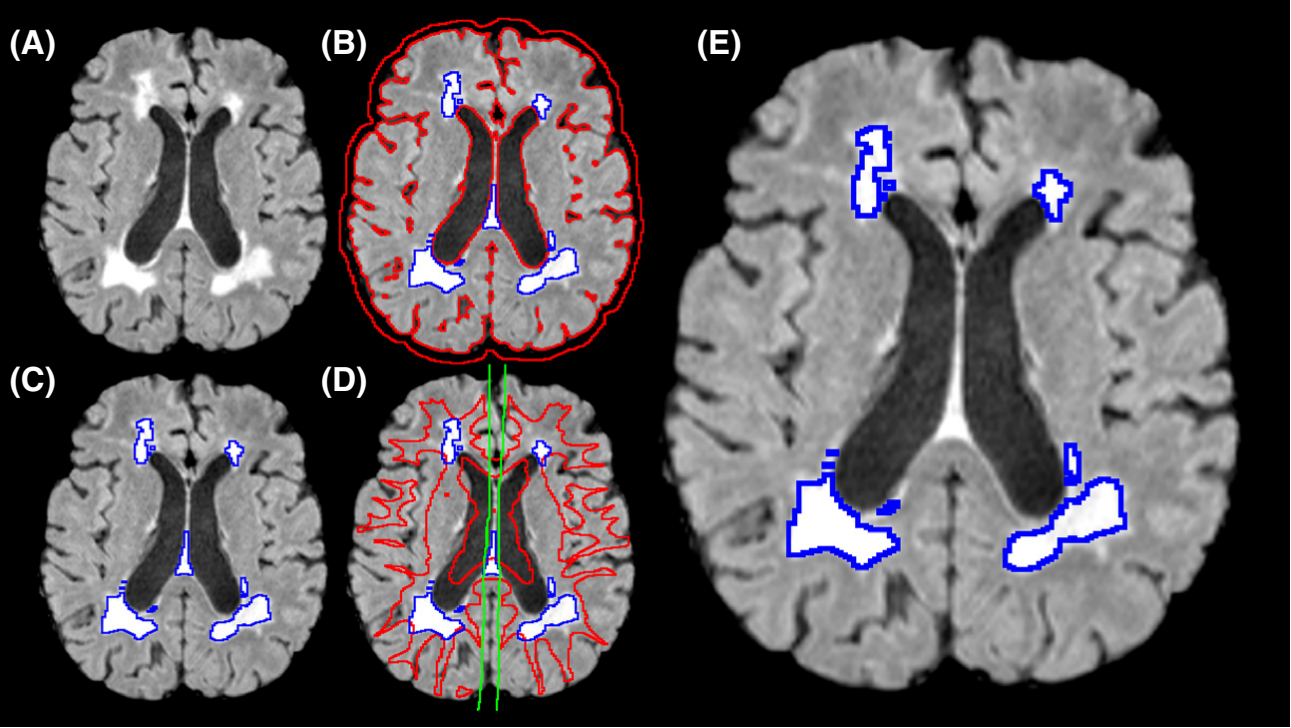
Illustration of lesion mapping method. (A) Representative axial slice, after brain extraction and smoothing. (B) Nonbrain mask from FAST segmentation. Bright hyperintensities (blue outline) are trivial to distinguish from dark CSF (red outline). (C) Nonbrain image (blue outline) has been thresholded to remove CSF from mask. (D) Lesions (blue outline) touching the mid‐sagittal mask (green outline) or not overlapping with the white matter mask (red outline) are removed. (E) Final lesion map (blue outline).

We developed a new, unsupervised, single‐image method for generating lesion masks and volumes, based on the commonly used open‐source software FSL. This software package is used in many neuroimaging labs to perform structural and functional image processing (Smith et al. 2004). In particular, we used the following tools: Brain Extraction Tool (BET) (Smith 2002a), FMRIBs Automated Segmentation Tool (FAST) (Zhang et al. 2001), FMRIBs Linear Image Registration Tool (FLIRT) (Jenkinson and Smith 2001; Jenkinson et al. 2002), FMRIBs Nonlinear Image Registration Tool (FNIRT) (Smith 2007), and FSLmaths. We scripted these tools to create a lesion map and T2LV from the FLAIR image (Fig. 1).

We started with skull stripping via BET on the FLAIR image. Then, the image was slightly blurred (*σ *= 0.5 mm) using FSLmaths to reduce false positives resulting from noise. Most central to our method was FAST, which uses Gaussian fitting of the intensity histogram to segment brain images by tissue type (gray matter (GM), white matter (WM), and nonbrain/CSF) (Zhang et al. 2001). The basis for our method stemmed from noticing that because voxels making up T2 hyperintensities are much brighter than other voxels in the brain, they lie outside the fitted Gaussian distribution of brain tissue on the histogram, and therefore were erroneously labeled as nonbrain/CSF. If one then looked only at the voxels classified as nonbrain, it was trivial to separate these hyperintense regions from CSF, blood, and dura, which are dark in this fluid‐attenuated sequence. We accomplished this by simply iteratively removing the darkest bin from the histogram until an empty bin was found. This bin value was then used to threshold the “nonbrain” voxels, removing true nonbrain tissue, leaving the hyperintensities.

This process sensitively marked lesions, but also erroneously marked the septum pellucidum, small regions of peripheral GM, and several midbrain GM structures that are consistently bright. We ran two additional steps to remove these. First, we created a FLAIR‐like standard space image by subtracting the ICBM CSF mask from the ICBM T2 standard mask. We used FLIRT (Jenkinson and Smith 2001; Jenkinson et al. 2002) and FNIRT (Smith 2007) with this standard to nonlinearly warp the ICBM WM mask to subject space. Voxels with WM probability above 0.7 were included in the mask. We then selected lesions that had at least one voxel within the WM mask, thereby eliminating false positives from outside the WM. Next, we removed midline false positives such as the septum pellucidum by eliminating any lesions that touch or come close (within 4 mm) to touching the mid‐sagittal plane. This was also accomplished with standard space masking. Because some subjects had periventricular lesions that were contiguous with the false‐positive septum, removal was truncated to a maximum distance from midline (9 mm), beyond which lesion‐labeled pixels were not removed. Final T2LV was expressed as a percentage of brain volume as calculated from FAST results.

#### Gray and white matter atrophy

Since we were interested in the ability of the estimated T2LVs from our method to uniquely correlate with disability status in MS, we compared the T2LVs with other MRI measures that have demonstrated success in predicting behavior and performance: gray matter volume (GMV) and white matter volume (WMV) (Grassiot et al. 2009). These measures were produced by the FSL (Smith et al. 2004) tool SIENAX, which we modified to accept the FLAIR image in addition to the T1‐weighted image, in order to improve delineation between brain matter and CSF. GMV and WMV were normalized to intracranial volume by multiplying by the ratio between each subject's intracranial volume and that of a standard. The result of this is a volume measure in standard space rather than subject space (normalized mm^3^) such that a smaller value corresponded to a smaller portion of the intracranial space, and therefore a greater degree of atrophy of that tissue type (Smith et al. 2002).

### Clinical outcomes

To determine the ability of our T2LVs to predict disease severity, we assessed its correlation with four commonly used measures of clinical disability: Expanded Disability Status Scale (EDSS), Six‐Minute Walk (6MW), Timed 25 Foot Walk (T25FW), and Symbol Digit Modalities Test (SDMT).

#### Timed 25‐foot walk

The T25FW is a measure of walking speed. The T25FW consisted of the participant walking 25 feet as quickly and safely as possible in a hallway clear of obstacles (Hobart et al. 2013). Two trials were performed, and the main outcome measure was mean speed, reported in feet/second (Goodman et al. 2009).

#### Six‐minute walk

The 6MW is a measure of walking endurance. It was performed in a rectangular, carpeted corridor with hallways exceeding 50 m length and clear of obstructions and foot traffic. We provided standardized instructions and emphasized walking as far and fast as possible for 6 min on a surface consistent with the original validation work in MS (Goldman et al. 2008). One researcher followed alongside for safety, while another followed 1 m behind recording distance traveled (feet) using a measuring wheel (Stanley MW50, New Briton, CT) (Motl et al. 2012); longer distances reflect better walking endurance (Goldman et al. 2008).

#### Expanded disability status scale

Expanded disability status scale is based on an evaluation of eight functional systems (FS), including visual, brainstem, pyramidal, cerebellar, sensory, bowel/bladder, cerebral, and other as well as ambulatory function (i.e., 500 m walk). The FS scores receive “step” scores which are combined with ambulatory function into an overall score. The EDSS score can range between 0 (no disability) and 10 (death from MS) (Kurtzke 1983).

#### Symbol digit modalities test

We included the Symbol Digit Modalities Test (SDMT) (Smith 2002b) as a measure of information processing speed (IPS) consistent with previous research; (Batista et al. 2012) the measure was administered by personnel who were not involved in the MRI acquisition or analyses. The oral response form of the SDMT provides a relatively quick assessment and is valid in persons with MS (Benedict et al. 2006). The SDMT captures visual/spatial processing speed and working memory. The main outcome measure of the SDMT was the total number of correctly provided numbers (maximum of 110) in the 90‐sec period (Smith 2002b; Benedict et al. 2006) with higher scores reflecting better IPS.

#### Statistics

Data analysis was performed using statistical package for the social sciences version 21.0 (SPSS, IBM Corp, Armonk, NY). All dependent variables (DV: EDSS, 6MW, T25FW, and SDMT) and independent variables (IV: age, WMV, GMV, and T2LV) were examined for normality and outliers by visual inspection of histograms and normal Q–Q plots. Non‐normal variables were transformed by square root, logarithmic, or inverse functions to result in a normal distribution, and outliers were defined as being at least three standard deviations from the mean.

Pearson correlations were computed between all variables. Age had significant correlation with all other DVs and IVs except SDMT, and so it was identified as a nuisance variable. To exclude the effects of age from further analysis, we examined partial correlations between the DVs and IVs, controlled for age. If no significant correlation existed between IV and DV after correction for age, then the pairing was dropped from further analysis.

We next performed hierarchical multiple regression (HMR) (Tabachnick and Fidell 2012) to answer two questions: First, is a significant amount of additional variance explained by adding T2LV to a model already containing the other significant IVs? Second, how much additional variance is explained when these other IVs are added to a model of age only? Examination of normalized ß coefficients in the final model also yielded the relative importance of each IV in explaining that clinical measure for MS.

## Results

**Figure 2:**
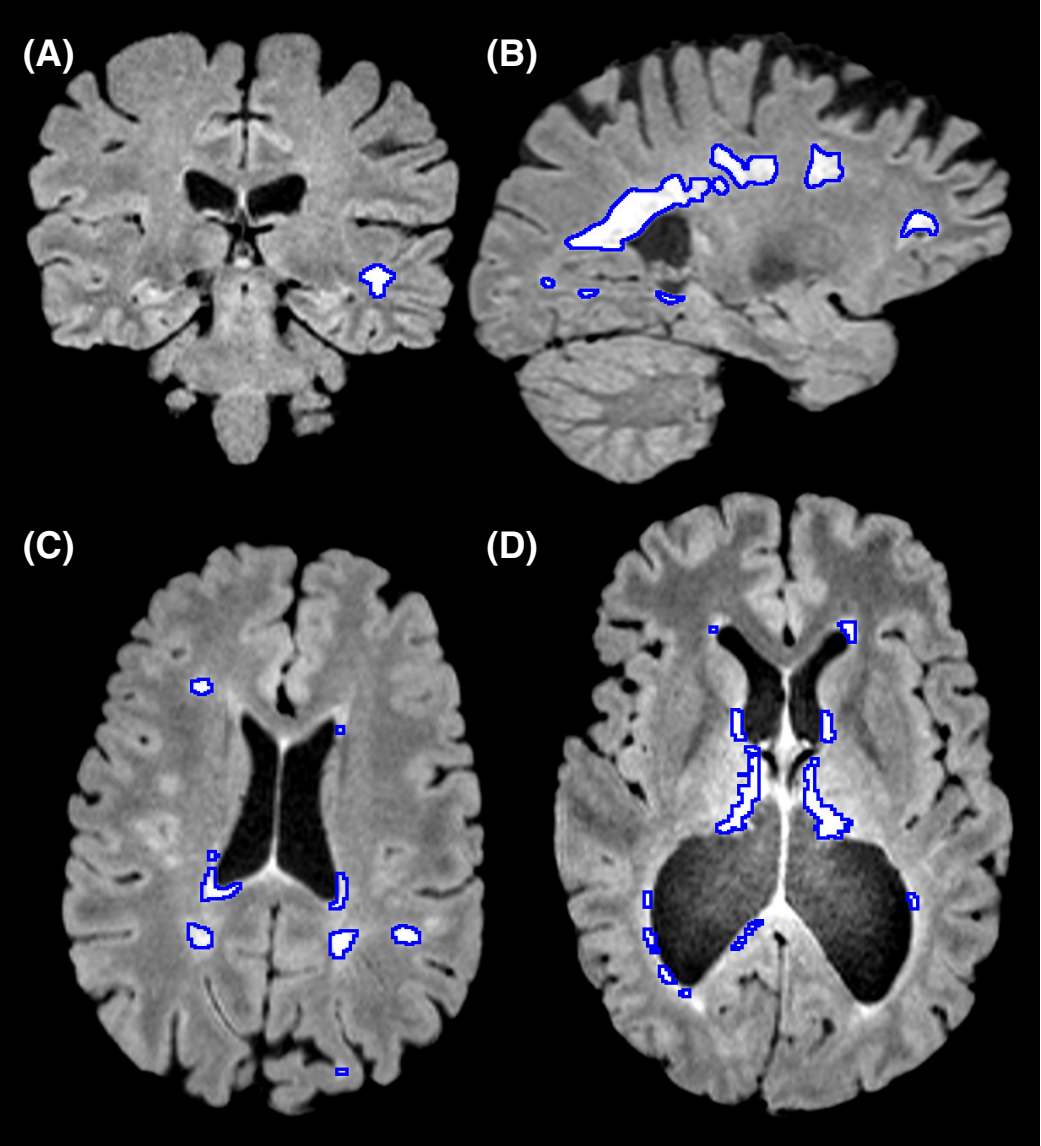
Representative examples of lesion mapping output (blue outline). (A) A coronal slice showing a lesion in peripheral white matter. (B) A sagittal slice showing large periventricular lesions. (C) An axial slice showing both periventricular and peripheral white matter lesions. (D) A case where severe atrophy caused midline false positives to not be removed, as they were further from midline than expected.

Our T2LV script ran successfully without human intervention on all 52 subjects. Average T2LV was 0.86% of total brain volume. Average WMV was 721,215 normalized mm^3^ and average GMV was 662,147 normalized mm^3^. Median EDSS was 5.5, while average 6MW, T25FW, and SDMT were 1131 feet, 3.87 feet/second, and 46 correct numbers, respectively (Table 1). Average computation time for our T2LV measurement was 3.6 h per subject with the nonlinear registration steps taking the majority of this time, at 2.5 h per subject. Figure 2 shows a few representative examples of output lesion maps.

### Statistical analysis

All measures were normally distributed except T2LV, which became normal after a log10 transform. One subject was removed from analysis as an outlier due to an SDMT score of 102, which was 3.8 standard deviations above the group mean. Significant Pearson correlations (Table 2) were found between all MRI and disability measures except GMV versus EDSS (*P* = 0.166) and GMV versus T25FW (*P* = 0.056). Age was significantly correlated with all MRI and disability measures except for SDMT (*P* = 0.068), so we examined partial correlations (Table 3) between DVs and IVs, controlled for age. MRI measures were still significantly correlated with each other, but GMV was no longer significantly correlated with any disability measure except SDMT (*P* = 0.006). WMV and T2LV continued to be significantly correlated with all disability measures except for EDSS (*P* = 0.051 and *P* = 0.060, respectively).

**Table 2:** Pearson correlation coefficients

Variable	1	2	3	4	5	6	7	8	9
1: LOG(T2LV)	1								
2: WMV	−0.532**	1							
3: GMV	−0.582**	0.693**	1						
4: EDSS	0.344*	−0.334*	−0.195	1					
5: 6MW	−0.513**	0.406**	0.324*	−0.779**	1				
6: T25FW	−0.438**	0.466**	0.267	−0.796**	0.917**	1			
7: SDMT	−0.499**	0.704**	0.442**	−0.511**	0.556**	0.588**	1		
8: Age	0.373**	−0.280*	−0.410**	0.289*	−0.378**	−0.404**	−0.255	1	
9: Gender	0.099	0.205	0.024	−0.110	0.070	0.054	−0.018	−0.134	1

LOG(T2LV), Log‐transformed T2 Lesion Volume; WMV, White Matter Volume; GMV, Gray Matter Volume; EDSS, Expanded Disability Status Scale; 6MW, 6 Minute Walk; T25FW, Timed 25 Foot Walk; SDMT, Symbol Digit Modalities Test.

Significance tests between groups (2‐tailed): *P < 0.05, **P < 0.01.

**Table 3:** Partial correlation coefficients, controlled for age

Variable	1	2	3	4	5	6	7
1: LOG(T2LV)	1						
2: WMV	−0.480**	1					
3: GMV	−0.507**	0.660**	1				
4: EDSS	0.266	−0.275	−0.087	1			
5: 6MW	−0.433**	0.338*	0.200	−0.756**	1		
6: T25FW	−0.392**	0.402**	0.121	−0.775**	0.903**	1	
7: SDMT	−0.450**	0.682**	0.382**	−0.472**	0.513**	0.548**	1

LOG(T2LV), Log‐transformed T2 Lesion Volume; WMV, White Matter Volume; GMV, Gray Matter Volume; EDSS, Expanded Disability Status Scale; 6MW, 6 Minute Walk; T25FW, Timed 25 Foot Walk; SDMT, Symbol Digit Modalities Test.

Significance tests between groups (2‐tailed): df = 49, *P < 0.05, **P < 0.01.

We performed HMR (Table 4) to examine the specific contribution of lesion volume on the disability metrics when controlled for other variables. EDSS was excluded because statistical significance was not achieved in the previous step. For 6MW, T25FW, and SDMT, a three‐stage regression was performed. The first model contained age as the only predictor. The second model added WMV for all three disability measures, as well as GMV for SDMT. T2LV was added in the final model. This revealed how much additional variance WMV (and GMV in the case of SDMT) accounts for after controlling for age, and then how much additional variance T2LV accounts for after controlling for all nuisance variables. ß coefficients were then examined in the final models to determine the relative importance of all predictors (Table 5).

**Table 4:** Hierarchical multiple regressions

	Model	Predictor variables	*R*	*R* ^2^	Adj. *R* ^2^	*R* ^2^ change	*F* change	Sig. *F* Change
6MW	1	Age	0.378	0.143	0.126	0.143	8.319	0.006
	2	+WMV	0.490	0.240	0.209	0.098	6.299	0.015
	3	+LOG(T2LV)	0.568	0.322	0.280	0.082	5.788	0.020
T25FW	1	Age	0.404	0.164	0.147	0.164	9.781	0.003
	2	+WMV	0.546	0.298	0.270	0.135	9.419	0.003
	3	+LOG(T2LV)	0.584	0.341	0.300	0.043	3.136	0.083
SDMT	1	Age	0.255	0.065	0.046	0.065	3.476	0.068
	2	+WMV, GMV	0.712	0.507	0.476	0.442	21.529	0.000
	3	+LOG(T2LV)	0.732	0.535	0.496	0.028	2.847	0.098

LOG(T2LV), Log‐transformed T2 Lesion Volume; 6MW, 6 Minute Walk; T25FW, Timed 25 Foot Walk; SDMT, Symbol Digit Modalities Test; WMV, White Matter Volume; GMV, Gray Matter Volume.

**Table 5:** Final models from multiple regressions

Dependent variable	Sig. (ANOVA)	Predictor	ß	Sig.
6MW	0.000	Age	−0.201	0.125
		WMV	0.163	0.255
		LOG(T2LV)	−0.351	0.020
T25FW	0.000	Age	−0.235	0.070
		WMV	0.264	0.063
		LOG(T2LV)	−0.255	0.083
SDMT	0.000	Age	−0.058	0.607
		WMV	0.713	0.000
		GMV	0.201	0.190
		LOG(T2LV)	−0.215	0.098

LOG(T2LV), Log‐transformed T2 Lesion Volume; WMV, White Matter Volume; 6MW, 6 Minute Walk; T25FW, Timed 25 Foot Walk; SDMT, Symbol Digit Modalities Test.

For 6MW, HMR showed that 14.3% (*P* = 0.006) of variance was explained by age alone, an additional 9.8% (*P* = 0.015) by adding WMV, and an additional 8.2% (*P* = 0.020) by adding T2LV. T2LV (ß = −0.255; *P* = 0.020) had the highest ß and was the only significant predictor of 6MW in the final model. The complete model accounted for 32.2% (28.0% adjusted, *P* = 0.000) of variance in 6MW.

For T25FW, HMR showed that 16.4% (*P* = 0.003) of variance was explained by age alone, with an additional 13.5% (*P* = 0.003) by adding WMV. Adding T2LV explained an additional 4.3%, but was not statistically significant (*P* = 0.083). The complete model accounted for 34.1% (*P* = 0.000).

For SDMT, 6.5% (*P* = 0.068) of variance was explained by age alone, but it was not significant. Adding WMV and GMV explained an additional 44.2% (*P* = 0.000). Adding T2LV explained an additional 2.8%, but this was not significant (*P* = 0.098). The complete model accounted for 53.5% (*P* = 0.000).

## Discussion

Quantification of lesions has been important in the diagnosis and monitoring of MS (Montalban et al. 2010; Polman et al. 2011), and as a measure of efficacy in drug trials (Sormani et al. 2010; Sormani and Bruzzi 2013), yet automated methods have been lacking. Further, by building our method upon widely used open‐source software, our technique will be highly accessible to neuroimaging and MS researchers.

In our analysis of the clinical utility of our T2LV measure, we observed that it was a significant predictor of clinically relevant disability measures. This indicates that our measure captured specific information about the disease state in our MS subjects. Significant correlations were observed between T2LV and metrics of clinical disability (Table 2) even when controlling for age (Table 3). The correlations of T2LV with the clinical and behavioral measures were all statistically significant with coefficients ranging from *r* = 0.266 to *r* = 0.513, except for EDSS after controlling for age (*P* = 0.060).

Though significant (*P* < 0.05) and highly significant (*P* < 0.01) Pearson correlations were found between T2LV and all predictors, significance levels decreased as we progressed to more advanced statistical analyses, controlling for age and brain volume measures. As T2LV was marginally significant (*P* < 0.10) in these later analyses, it is possible that significance could be demonstrated in a future study with a larger sample size. It can be difficult to show statistical significance in multivariate analyses with underpowered preliminary studies such as ours (Tabachnick and Fidell 2012). Despite the limited number of subjects, we did find significance in the predictive power of T2LV on 6MW even when controlling for all other variables, showing promise for future, larger studies.

It is important to note that white matter atrophy is also highly associated with disability in MS, as we have shown with our WMV measure. We note that our WMV measure was not the standard output that would be obtained by running SIENAX in FSL, but instead, we leveraged both the T1 and T2 images in order to segment brain and identify GM/WM borders. In addition, our WMV measure benefits from our lesion mapping as an accurate lesion mapper is required in order to restore volume to WM that was misclassified as GM due to the MS lesions. With these corrections, we obtained an impressive predictive power for white matter volume: we saw a ß coefficient of 0.713 with significance *P* = 0.000 in the final model for SDMT with three other predictor variables. This further demonstrates the value of our lesion mapping method, as the change in contrast of lesions on T1‐weighted imaging means good lesion maps are required for accurate measures of gray and white matter volumes.

This method in its current form is not without drawbacks. There are three parameters (WM probability threshold, and two distance measures related to midline false‐positive removal) whose default values were chosen based on manual inspection of a relatively small number of images. If desired, these parameters can be set to other values at runtime, which can affect the sensitivity and specificity of the result. As an example, Figure 2D shows a case where midline false‐positives were not removed because severe ventricular enlargement caused them to appear further from the midline than expected. Future work could include more rigorous tuning of these parameters, or devising a way to automatically tune them for each subject based on that subject's specific anatomy, which could further improve associations with disability. Also, though obtaining T2LV from a single FLAIR image is cost‐effective, convenient, and useful, FLAIR is known to be less sensitive to lesion detection in the posterior fossa (Filippi et al. 2011). Future modification to our method to include analysis of a non‐FLAIR T2‐weighted image could potentially improve correlations.

Despite these caveats, the correlation our T2LV measure achieved with EDSS (*r* = 0.344, *P* = 0.013) compares favorably with previously published studies, which have produced correlations from 0.19 to 0.47 (Mammi et al. 1996; Gawne‐Cain et al. 1998; Bonneville et al. 2002; Bonati et al. 2011; Cohen et al. 2012; Kearney et al. 2014), with one study failing to achieve a significant correlation (Miki et al. 1999). It is interesting to note that the studies that reported simple correlations chose to report Spearman coefficients, while we have reported Pearson coefficients. Spearman coefficients are generally higher because, based on rank order, they are not penalized by lack of a linear relationship. Our use of Pearson correlations, which indicate the degree of linear relationship between two variables, is more conservative and supports stronger conclusions.

Our method proceeded automatically without requiring manual intervention. While fully automated methods for T2LV measurement have been previously described (Mortazavi et al. 2011; Lladó et al. 2012), none of these studies reported correlations with clinical metrics to evaluate the sensitivity of the methods to disease. Previous studies that reported correlations with clinical metrics have instead used time‐consuming methods that were at least partially manual (Gawne‐Cain et al. 1998; Miki et al. 1999).

## Conclusion

T2LV in persons with MS has been a hallmark feature of clinical diagnosis and tracking of disease progression and the effectiveness of clinical interventions, indicating the need for an accessible tool to better facilitate its measurement. We have created an intuitive, fully automated lesion mapping, and quantification package based on the open‐source, readily available neuroimaging software package FSL. To the best of our knowledge, we have provided the first fully automated package that requires only a single image, a 3D FLAIR. We have validated the method by demonstrating its ability to predict clinical disability. To the best of our knowledge, this is the only fully automated tool validated in this way. We have made our package and its source freely available (http://mrfil.bioen.illinois.edu) in hopes that it will lower costs and enable better comparability across studies using modern high‐resolution FLAIR MRI of MS subjects and in normal aging.

## Conflict of Interest

The authors declare that there is no conflict of interest.
